# Bio-Based Phenolic Aldehyde Functionalization of Cellulose Acetate–Polyethyleneimine Membranes for Enhanced Ni^2+^ and Cu^2+^ Retention

**DOI:** 10.3390/polym18182193

**Published:** 2026-09-08

**Authors:** Eduard Ionut Piscanu, Celina Maria Damian, Andreea Madalina Pandele, Madalina Oprea, Adrian Ionut Nicoara, Stefan Ioan Voicu

**Affiliations:** 1 Department of Analytical Chemistry and Environmental Engineering, Faculty of Chemical Engineering and Biotechnologies, National University of Science and Technology POLITEHNICA Bucharest, 1-7 Gheorghe Polizu, 011061 Bucharest, Romania; eduard.piscanu@yahoo.com (E.I.P.); pandele.m.a@gmail.com (A.M.P.); madalina.calarasu@upb.ro (M.O.); 2 Advanced Polymer Materials Group, National University of Science and Technology POLITEHNICA Bucharest, 1-7 Gheorghe Polizu, 011061 Bucharest, Romania; celina.damian@upb.ro; 3 Department of Science and Engineering of Oxide Materials and Nanomaterials, Faculty of Chemical Engineering and Biotechnologies, National University of Science and Technology POLITEHNICA Bucharest, 1-7 Gheorghe Polizu, 011061 Bucharest, Romania; adi.nicoara18@gmail.com; 4 Academy of Romanian Scientists, 54 Splaiul Independentei Street, District 5, 050094 Bucharest, Romania

**Keywords:** water treatment, polymeric membrane, functional membrane, bio-based phenols, cellulose acetate membrane

## Abstract

The increasing occurrence of heavy metal ions in water and wastewater streams represents a serious concern for both the environment and human health. The efficient removal of such contaminants requires the development of stable and functional membrane materials capable of combining separation performance with specific metal-binding interactions. This work proposes the use of bio-sourced phenols alongside branched polyethyleneimine and cellulose acetate to develop advanced membranes for the retention of Ni^2+^ and Cu^2+^ ions from aqueous solutions. The chemical modification of the cellulose acetate membrane was confirmed by structural and thermal analysis. Improved thermal resistance between 50–200 °C suggests that chemical interactions as well as hydrogen bonds were developed within the functionalized membranes. The effect of aldehyde modification on membrane chemistry, morphology, thermal behavior, mechanical properties, and filtration performance was systematically investigated. The vanillin-modified membrane showed the best mechanical response, likely due to improved matrix cohesion promoted by its methoxy-substituted aromatic structure. In contrast, the salicylaldehyde-modified membrane exhibited the highest metal-ion retention, reaching approximately 73% for Ni^2+^ and 67% for Cu^2+^ after five filtration cycles. These findings highlight the potential of bio-based phenolic aldehydes as active compounds for designing membranes with tailored morphology, stability, thermal, mechanical, and metallic ion-removal performances.

## 1. Introduction

Over the last few years, accelerated urbanization and the growth of technological and industrial activities have significantly contributed to water pollution and contamination [1,2,3]. In addition to conventional pollutants, modern water and wastewater streams now contain contaminants that can have a high impact on the environment and human health even in low concentrations. The most representative compounds from this category are heavy metal ions [4]. This type of pollutant, especially Cu^2+^, Ni^2+^, Pb^2+^, Cd^2+^, and Zn^2+^ ions that may originate from mining, electroplating, battery production, electronics, and pigment manufacturing, are amongst the most dangerous as they are non-biodegradable, tend to accumulate in ecosystems and can pose risks to human health and aquatic life even at low concentrations [5,6,7].

Conventional water purification technologies such as precipitation, coagulation–flocculation, ion-exchange, and adsorption have been widely investigated for the retention of heavy metal ions [8,9,10]; however, these techniques lack efficiency, have poor selectivity, and imply high chemical reagent consumption. Additionally, sludge generation has imposed the necessity to develop novel materials that possess structural stability, high water permeability, and specific metal-binding affinity.

Membrane separation represents one of the most used techniques in water purification due to its ability to combine high efficiency with broad applicability over a wide range of industries [11]. Membranes can be used in continuous systems and can be tailored to achieve targeted separation through various mechanisms. While conventional polymeric membranes rely mostly on physical separation, the inclusion of functional groups that are capable of interacting with the metallic ions can lead to the development of active separation platforms [12,13,14,15].

Cellulose acetate is one of the most used cellulose derivatives for membrane synthesis due to its high processability, good film-forming capacity, and hydrophilic character. Its rich oxygen-containing functional-group backbone, incorporating ester and residual hydroxyl, plays a crucial role in further modification. These functional groups play a crucial role in water affinity and provide numerous sites for intermolecular interactions. Nevertheless, cellulose acetate generally exhibits limited affinity and selectivity towards specific heavy metal ions, which imposes a need for further functionalization [16,17,18].

Branched polyethyleneimine is a versatile functional polymer bearing a high density of primary, secondary, and tertiary amino functionalities that can actively participate in the development of strong interactions with metallic ions [19,20,21]. These nitrogen-containing sites can interact with metallic ions through coordination or electrostatic interactions, making PEI a valuable component for the development of adsorptive membranes. When incorporated into cellulose acetate, PEI can enhance the metal-binding performance of the membrane due to its amino-rich backbone [22,23]. Luo and collaborators developed fibrous electrospun membranes based on cellulose acetate and polyethyleneimine immobilized by cross-linking for the removal of Cr (IV) [24]. Results demonstrated that the developed system can efficiently reduce the concentration of Cr(VI) to 0.03 mg/L from the initial concentration of 50 mg/L, achieving concentrations below drinking-water guideline limits.

However, the high hydrophilicity and partial water solubility of PEI may lead to leaching or poor stability under aqueous filtration conditions. Therefore, stabilization of the PEI phase is necessary in order to obtain membranes that are capable of withstanding repeated water-treatment processes. One of the most common approaches is to crosslink this polymer to increase its stability, and one of the most applied strategies is aldehyde crosslinking [25]. In the cellulose acetate PEI system, glutaraldehyde, as a bifunctional crosslinker, can react with the amino groups of PEI through Schiff base formation, leading to imine-type linkages and partial network development.

Phenolic aldehydes represent an attractive class of bio-based compounds for polymer systems due to their reactivity and functional versatility [26]. Compounds such as vanillin and salicylaldehyde contain aldehyde groups capable of reacting with amino functionalities through Schiff base formation, while their aromatic and phenolic structures can introduce additional hydrogen-bonding, polar, and metal-interaction sites [27,28,29,30]. In CA/PEI membranes, this dual functionality is particularly relevant, as the aldehyde group can contribute to both the stabilization and functionalization of the PEI-rich phase, whereas the phenolic oxygen-containing groups may participate in secondary interactions and potentially enhance the affinity toward metal ions. Therefore, the use of vanillin and salicylaldehyde offers a sustainable strategy to tune both the structure and functionality of CA/PEI membranes for water purification applications.

In this context, the present study reports the preparation of cellulose acetate–polyethyleneimine membranes stabilized with glutaraldehyde and modified with vanillin or salicylaldehyde for the retention of Ni^2+^ and Cu^2+^ ions from aqueous solutions. The novelty of this work lies in the use of bio-based phenolic aldehydes as functional modifiers for CA/PEI membranes, enabling their molecular architecture to influence membrane chemistry, porous architecture, thermal and mechanical behavior, and metal-ion retention. By comparing vanillin and salicylaldehyde, this study provides insight into how specific aldehyde-derived functionalities, such as methoxy-substituted aromatic groups or ortho-phenolic N/O coordination environments, can tune the performance of amine-rich membranes for water purification applications.

## 2. Materials and Methods

### 2.1. Materials

Cellulose acetate powder (CA) (average Mn ~30,000, determined by GPC), branched polyethyleneimine (PEI) (average Mw ~800 by LS, average Mn ~600 by GPC), salicylaldehyde (SAL) (reagent grade, 98%), vanillin powder (VAN) ≥ 99.0%, and glutaraldehyde solution 25% (GA) (Aqueous ≥98%) from Sigma-Aldrich, Saint Louis, Missouri, United States were used as received. N,N-dimethylformamide (DMF, 99%,) and ethanol (≥99.8% GC) were used as solvents for polymer dissolution and functionalization. For the heavy metal retention tests, nickel(II) acetate tetrahydrate 98% (Ni(C_2_H_3_O_2_)_2_ × 4H_2_O) and copper(II) sulfate pentahydrate (CuSO_4_ × 5H_2_O), ACS reagent, ≥98.0% from Sigma Aldrich, Saint Louis, MO, USA were employed. For all experiments, distilled water was employed.

### 2.2. Membrane Synthesis

Cellulose acetate solution of 12 wt% concentration in dimethylformamide (DMF) was obtained under stirring at 50 °C for 24 h. After complete dissolution, the solution was cooled to room temperature, and PEI was incorporated under vigorous stirring. PEI was added at 20 wt% with respect to the CA mass. The resulting CA-PEI (**CAP**) solution was subsequently used for the synthesis of membranes. The amount of reagents are presented in Table 1.

Polymeric membranes were synthesized by casting the CA-PEI solution onto a glass plate with the aid of an automatic film applicator (Elcometer 4340, Manchester, UK; samples of 10 × 10 cm surface area and approximately 200 μm thickness were synthesized), followed by immersion in a coagulation bath (500 mL of distilled water at room temperature). The resulting membranes were then removed from the bath, washed with ethanol and distilled water in order to remove residual DMF, and subsequently dried in a vacuum oven at 40 °C for 24 h. The as-obtained membranes will henceforth be referred to as CAP. CAP was subsequently used as a base material for the subsequent synthesis of modified membranes through the incorporation of different functional compounds and crosslinking. A mixture of water–ethanol (70:30, *v*/*v*) was used as the reaction medium to preserve the structural integrity of the CA/PEI membranes while ensuring adequate solubility and diffusion of the aldehydes during the modification process:

- CAPG was obtained by immersing a CAP membrane in a GA solution for 30 min at room temperature. The crosslinking bath was placed on an orbital shaker and was prepared by solubilizing the GA (10 mM) in a mixture of water and ethanol (70:30). After the reaction was completed, CAP samples were thoroughly washed with the water/ethanol mixture and dried.
- CAPVG was prepared by immersing the CAP sample in a mixture of water and ethanol (70:30) containing vanillin (20 mM). The samples immersed in the vanillin bath were placed on an orbital shaker for 2 h at room temperature. After the completion of the reaction, the vanillin-modified membranes were extracted from the bath and immersed for 30 min in a crosslinking bath containing GA (10 mM) solubilized in a water–ethanol mixture. After the reaction was completed, CAPVG samples were thoroughly washed with the water/ethanol mixture and dried.
- CAPSG was prepared by immersing the CAP sample in a mixture of water and ethanol (70:30) containing salicylaldehyde (20 mM). The samples immersed in the salicylaldehyde bath were placed on an orbital shaker for 2 h at room temperature. After the completion of the reaction, the salicylaldehyde-modified membranes were extracted from the bath and immersed for 30 min in a crosslinking bath containing GA (10 mM) solubilized in a water–ethanol mixture. After the reaction was completed, CAPSG samples were thoroughly washed with the water–ethanol mixture and dried.

The initial reaction of PEI with vanillin or salicylaldehyde may partially reduce the number of free amino groups available for subsequent glutaraldehyde crosslinking. Therefore, the final network structure likely results from the combined effects of Schiff base formation with the phenolic aldehydes and glutaraldehyde-mediated crosslinking. Nevertheless, all membranes were treated under identical glutaraldehyde conditions, allowing the influence of the aldehyde structure on the membrane properties to be comparatively evaluated.

**Figure 1:**
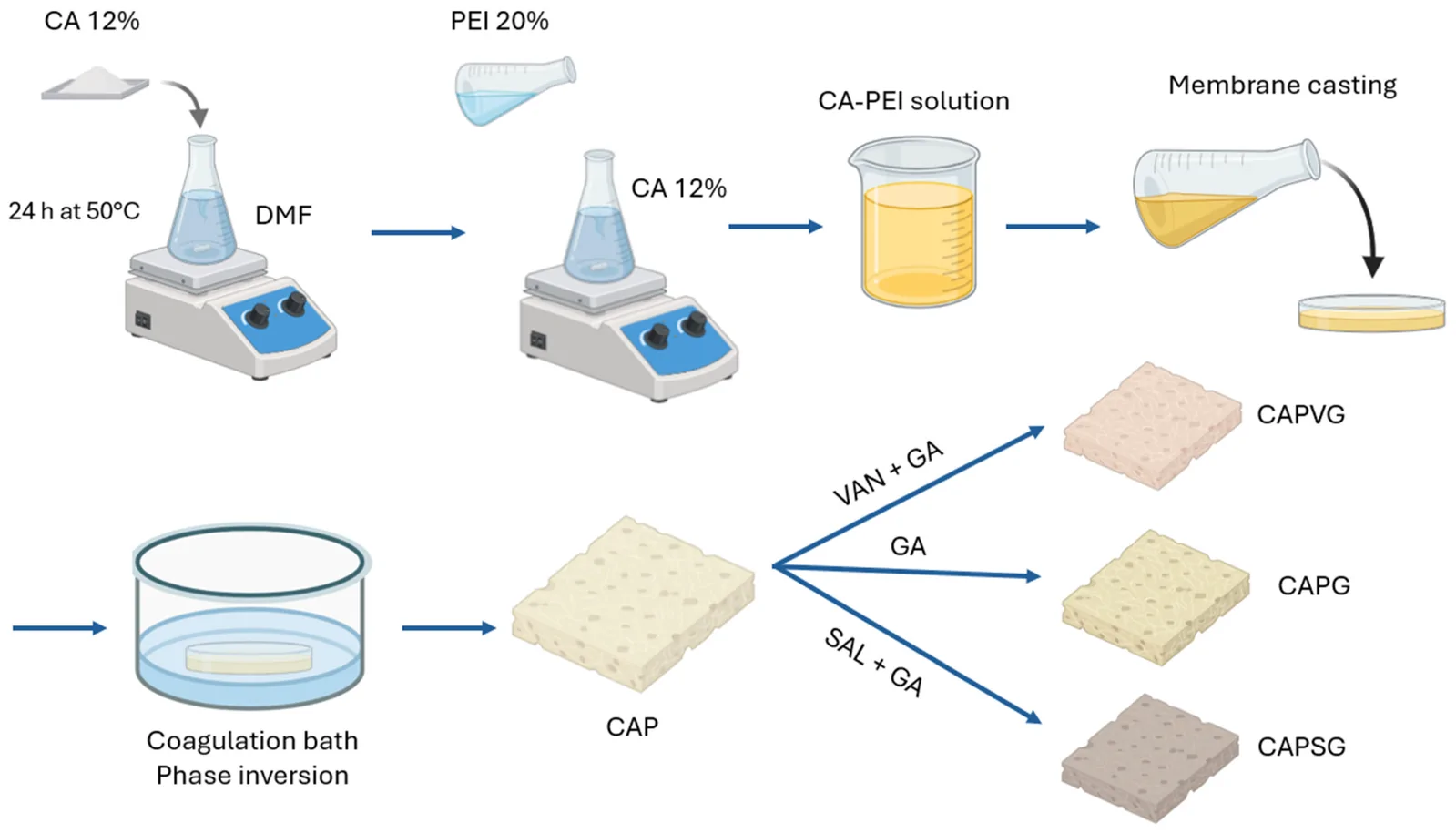
Schematic representation of the main steps followed in the synthesis of functional membranes.

Since vanillin and salicylaldehyde were introduced prior to GA crosslinking, their reaction with PEI may reduce the fraction of amino groups subsequently available for reaction with GA. Therefore, the differences between CAPG, CAPVG, and CAPSG reflect the combined effect of phenolic aldehyde incorporation and the resulting changes in the final crosslinked network. CAPG was consequently used as the GA-crosslinked reference without phenolic modification. Schematic representation of synthesis strategy is presented in Figure 1.

**Figure 2:**
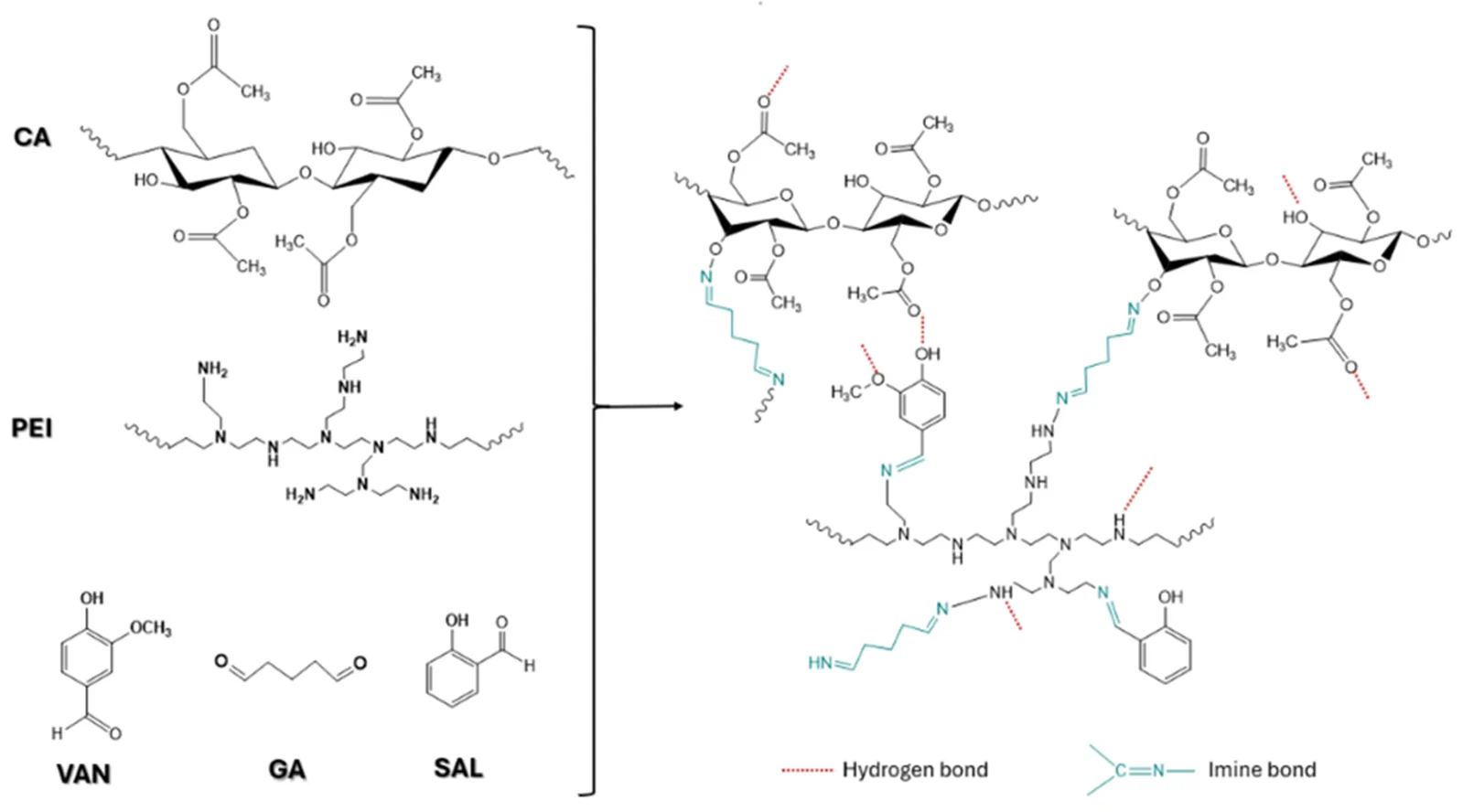
Schematic representation of the main interactions that may take place between the components of the CA-PEI aldehyde membranes.

Possible reaction mechanisms involved in the synthesis of modified membranes are schematically represented in Figure 2. The formation of the modified CA/PEI membranes may involve several simultaneous interactions between the system components. PEI can interact with cellulose acetate mainly through hydrogen bonding and polar interactions between its amino groups and the carbonyl, ether, and residual hydroxyl groups of CA. Glutaraldehyde is expected to react preferentially with PEI amino groups through Schiff base formation, generating imine linkages and partially stabilizing the amine-rich phase [25]. Vanillin and salicylaldehyde may further react with remaining PEI amino groups through similar aldehyde–amine reactions, while their phenolic hydroxyl, aromatic, and, in the case of vanillin, methoxy groups can introduce additional hydrogen-bonding and polar interactions [31]. In the salicylaldehyde-modified system, the ortho-phenolic hydroxyl group may also contribute to N/O-type coordination environments relevant for metal-ion binding.

### 2.3. Characterization

Fourier Transform Infrared Spectroscopy (FTIR) spectra were recorded on a Bruker Vertex 70 spectrometer (Bruker Optics GmbH & Co. KG, Ettlingen, Germany) in the 400–4000 cm^−1^ range with 4 cm^−1^ resolution and 32 scans. The samples were analyzed on the attenuated total reflection (ATR) module.

X-ray photoelectron spectrometry (XPS) analysis was performed on a K-Alpha spectrometer (Thermo Fisher Scientific, East Grinstead, UK) with a monochromatic Al Kα source (1486.6 eV) in a vacuum base pressure of 2 × 10^−9^ mbar. Charging effects were compensated using a flood gun, and binding energy was calibrated by placing the C1s peak at 284.8 eV as internal standard. Deconvolution of C1s signals was performed through a smart background algorithm with a convolved Gaussian–Lorentzian ratio. The pass energy for the survey spectrum was set at 200 eV, while for the high-resolution C1s spectra, it was 20 eV.

Thermogravimetric analysis (TGA) was performed using a Netzsch TG 209 F1 Libra analyzer (NETZSCH-Gerätebau GmbH, Selb, Germany), from RT to 700 °C under a nitrogen atmosphere with a heating rate of 10 °C/min.

Differential scanning calorimetry (DSC) curves were recorded on a Netzsch DSC 204 F1 Phoenix calorimeter (NETZSCH-Gerätebau GmbH) using a heating–cooling program from RT to 300 °C at a heating rate of 10 °C/min under a nitrogen atmosphere (20 mL/min flow rate).

Scanning electron microscopy (SEM) was performed using a Quanta Inspect F microscope (Thermo Fisher Scientific, Hillsboro, OR, USA), equipped with an energy-dispersive X-ray spectrophotometer (EDX), with an accelerating voltage of 30 kV.

Membrane performance in terms of water flux and metallic-ion retention was measured using a dead-end filtration setup at room temperature. Water flux measurements were performed by passing 100 mL through 47 mm diameter membrane disks at a transmembrane pressure of 0.8 bar; the feed solution was recirculated 5 times during the measurement. For metallic ions, retention solutions of 1 g/L for both ions were prepared. UV-Vis analysis was performed on feed solutions before and after circulation through the membrane at wavelengths of λ = 742 nm (for Cu^2+^) and λ = 392 nm (for Ni^2+^) using a Shimadzu UV-3600 UV-VIS spectrometer (Shimadzu Corporation, Kyoto, Japan) equipped with a quartz cell having a light path of 10 mm, using a calibration curve (2–10 μg/mL). All experiments were performed in triplicate, and the results are presented as mean ± standard deviation (SD).

Retention efficiency (R%) was calculated based on the following equation:

R (%) = (Cf − Cp)/Cf × 100(1)

where Cf is the solute concentration in the feed (mol·L^−1^), and Cp is the solute concentration in the permeate (mol·L^−1^).

The water flux (*Wf*) expressed in L·m^−2^·h^−1^ is calculated by the following equation:

(2) $$Wf=\frac{V}{A\cdot t}$$

where *Wf* is the water flux, *V* is the volume (L) of the feed solution, *A* is the area (m^2^) of the membrane, and *t* is the time (h).

The computed tomography (CT) scans of the plasma-treated membranes were obtained using a SkyScan™ 2211 from Bruker (Bruker, Billerica, MA, USA), equipped with an X-ray tube, at a tension of 30 kV and an intensity of 500 µA. The analysis was performed with a pixel dimension of 0.3 µm and a resolution of 2688 × 4032. The rotation step was fixed at 0.1 degrees, and charge-coupled device (CCD) scanning mode was used. The three-dimensional reconstruction was realized using the NRecon software, version 1.7.1.6. The 3D images were visualized using CTVox 2.1, and Data Viewer 2.1.0 was used to observe certain bi-dimensional architectural details.

Mechanical tests were performed on wet samples using a universal testing machine (Instron, Model 3382, Norwood, MA, USA) at a relative humidity of 50% and a speed of 2 mm/min. The size of the samples was 10 cm × 1 cm. At least six specimens were tested for each membrane composition, and the average values are reported. Young’s Modulus was calculated from the slope of the linear portion of the stress-strain curve.

Contact angle (CA) measurements were performed with the aid of a Drop Shape Analyzer-DSA100 from Krüss Scientific GmbH (Hamburg, Germany), using water at room temperature by the sessile drop method. The contact angle values were determined using the Young–Laplace equation in Advance software. Contact angle values were calculated from 20 measurements performed at each of three different positions on the membrane surface. The results are expressed as mean ± standard deviation.

## 3. Results

### 3.1. Structural Characterization

**Figure 3:**
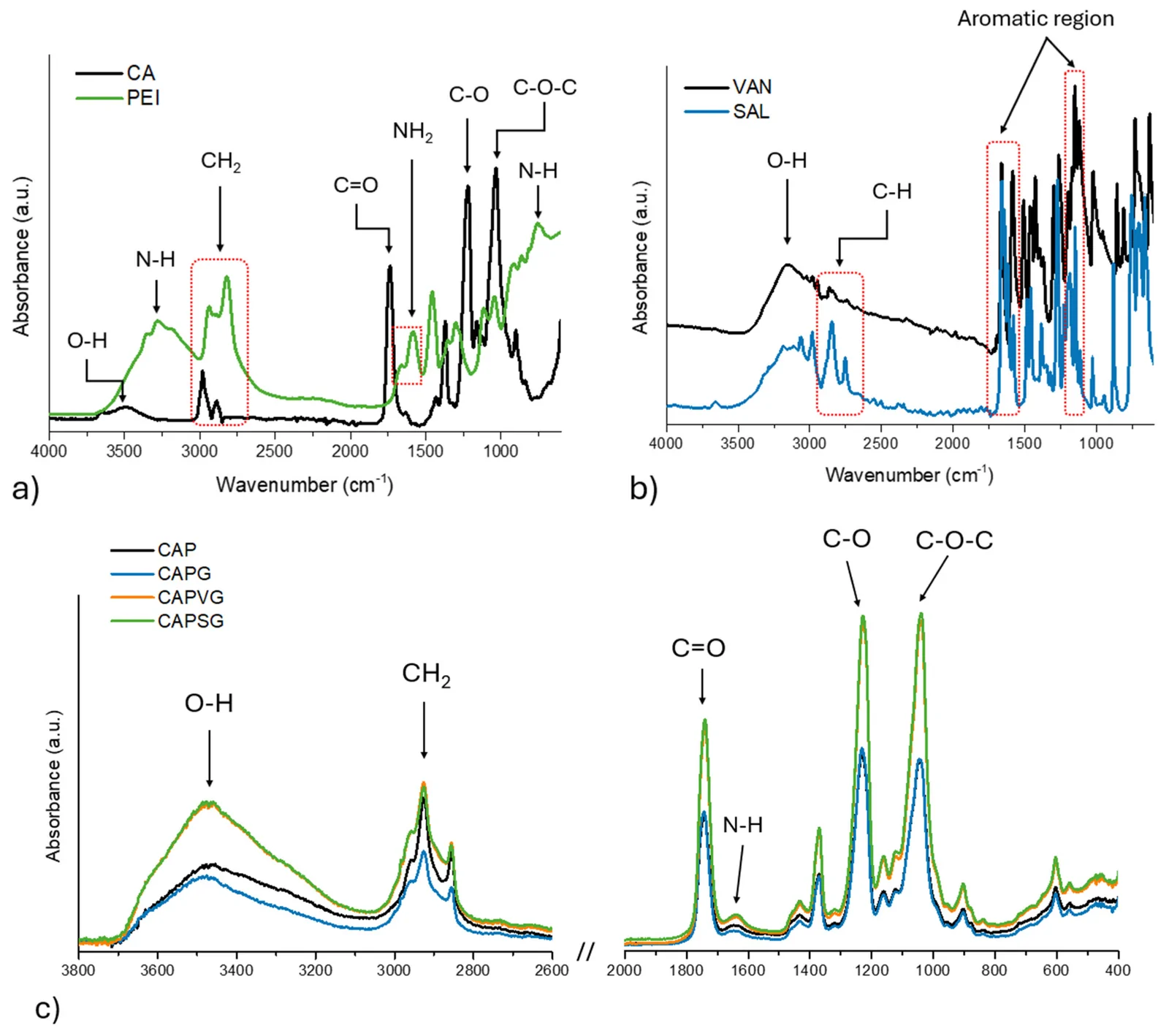
FTIR spectra of the neat materials used in the development of the membranes (**a**,**b**) and the corresponding membranes (**c**).

The chemical structures of the synthesized membranes, along with the materials used in the synthesis, were evaluated through FTIR, and the corresponding spectra are presented in Figure 3. CA sample shows distinct signals such as the broad band centered around 3500 cm^−1^ corresponding to O-H stretching vibrations and the peak at 1740 cm^−1^ characteristic of the carbonyl group, as well as the strong absorption bands from 1220 cm^−1^ and 1035 cm^−1^ assigned to the stretching vibrations of the acetyl group and C-O-C bonds [32]. The amino-enriched backbone of PEI is highlighted by several characteristic peaks such as ~3300 cm^−1^, which corresponds to the NH stretching vibration; 1586 cm^−1^ and 1664 cm^−1^, which are associated with the bending vibrations of NH_2_ groups; and 1117 cm^−1^ and 1043 cm^−1^, which correspond to C-N stretching vibrations [33].

Bio-sourced phenols, vanillin, and salicylaldehyde share a similar chemical structure characterized by phenolic hydroxyl stretching vibration around ~3200 cm^−1^ [34], an aromatic backbone (1500–1600 cm^−1^), and an aldehyde group characterized by the peaks from ~2800–2900 cm^−1^ (C-H from aldehyde) and 1670 cm^−1^ (C=O) [35].

Analyzing the CAP and CAPG samples, one can observe that, along with the glutaraldehyde crosslinking process, the peak band from 3200–3650 cm^−1^ displays a reduced intensity [24], confirming the decrease in the number of free amino groups as well as hydroxyl. For the membrane samples, the peak at ~3500 cm^−1^ becomes more intense with the inclusion of phenolic compounds due to hydrogen bonding between the multiple amino functionalities from PEI and phenolic OH as well as hydroxyl groups from the CA backbone [36]. Moreover, the increased intensity of the cluster peak between 2850–2980 cm^−1^ may be attributed to the enhanced contribution of aliphatic C-H stretching vibrations from PEI and glutaraldehyde-derived segments, together with possible contributions from aldehydic C-H vibrations when phenolic aldehydes are present.

The peak at 904 cm^−1^ can be assigned to overlapping contributions from the CA backbone and the amine-rich PEI oligomer and may suggest possible intermolecular interactions between the components. The imine bonds (C=N) formed during the reaction between the aldehyde groups from GA, vanillin, salicylaldehyde, and PEI are demonstrated by the signal at 1604 cm^−1^ [37,38,39]. In addition to the Schiff base formation, the additional phenolic OH as well as the methoxy group can form additional hydrogen bonds with both CA and PEI. This can be associated with the broadening of the signal from 3200–3600 cm^−1^.

Additionally, the small peak at 838 cm^−1^ may arise due to the oxygen-based linkages formed during aldehyde treatment, such as hemiacetal- or acetal-like structures, although the formation of specific hemiacetal or acetal linkages cannot be confirmed solely based on FTIR analysis [39].

**Figure 4:**
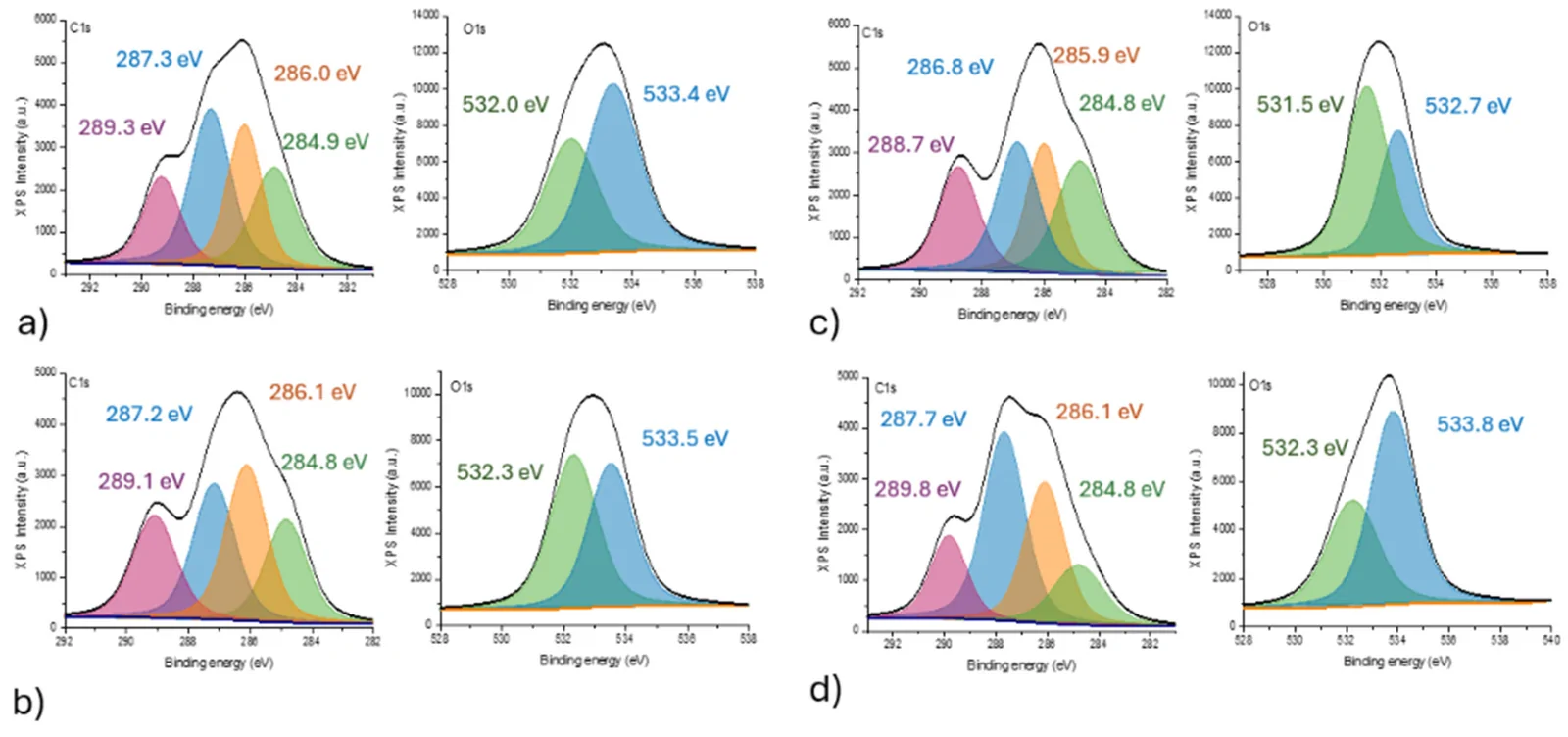
Deconvoluted high-resolution XPS spectra of C 1s and N 1s regions for CAP (**a**), CAPG (**b**), CAPVG (**c**), and CAPSG (**d**) samples.

The surface elemental composition of the synthesized membranes was evaluated through XPS analysis, and the corresponding results are presented in Figure 4 and Table 2. The survey spectra (Figure S2) for all samples confirmed the presence of C, O, and N as the main constituent elements. C and O are consistent with the chemical structure of the CA matrix and aldehyde components, while N is related to the incorporation of PEI. The preservation of part of this signal after aldehyde crosslinking confirms the existence of free amino groups from the PEI structure, as was further proven by a high-resolution N 1s spectrum (399.7 eV peak) (Figure S1).

The C-C/C-O ratio from the deconvolution of C 1s can be an indicator of possible changes that take place in the surface chemical composition of the membranes after the inclusion of aldehyde modifiers. Thus, one can observe that in the case of CAPG and CAPSG samples, lower values were determined. This may suggest an enrichment of oxygen functionalities at the membrane surface. This is consistent with the crosslinking process as well as possible structural changes or interfacial organization induced by the functionalization process. Conversely, the increased value computed for CAPVG may indicate that, in this case, the surface may be enriched in aromatic carbon species. The significant differences between the SAL and VAN samples can be correlated with their chemical structures. Salicylaldehyde has a phenolic group on the ortho position that may form strong hydrogen bonds, while vanillin has an additional methoxy group, which introduces C-O-containing species that may favor a different surface organization.

High-resolution deconvolution of the C 1s, O 1s, and N 1s regions provides an in-depth understanding of the chemical features of the membranes. For C 1s species, all spectra display four secondary peaks: C-C/C-H (284.8 eV), C-O (286.1 eV), and O-C=O from the ester groups of cellulose acetate (288.9 eV), which correspond to the chemical structure of the CA-PEI matrix [17,40].

When looking at the N 1s high-resolution spectra, it can be observed that after glutaraldehyde crosslinking, the initial 400.1 eV peak shifted toward higher values of binding energies as the curing process advanced. Moreover, the addition of V and S aldehyde components moved the position of nitrogen species towards 401.7 eV, confirming supplementary bridges with the PEI matrix.

The O 1s spectrum shows two types of oxygen species, highlighted by the existence of two secondary peaks. These correspond to O-C (532 eV) and O-C=O (533 eV). However, in the case of the CAPVG sample, the peak at 531 eV may arise from the participation of the oxygen in O=C/O=C-N bonds [41].

Due to the complexity of the CA/PEI–aldehyde-modified membranes, overlapping XPS contributions are expected. In the C 1s region, C-O species from cellulose acetate typically appear around 286.0–286.7 eV, while C-N from PEI and possible imine-related C=N contributions may occur in a close range, approximately 286.0–287.0 eV. Higher-binding-energy components around 287.5–289.0 eV are generally associated with carbonyl and ester environments. Therefore, C-N and imine-related signals may overlap with existing C-O/C=O contributions [42].

### 3.2. Thermal and Mechanical Properties of the Membranes

**Figure 5:**
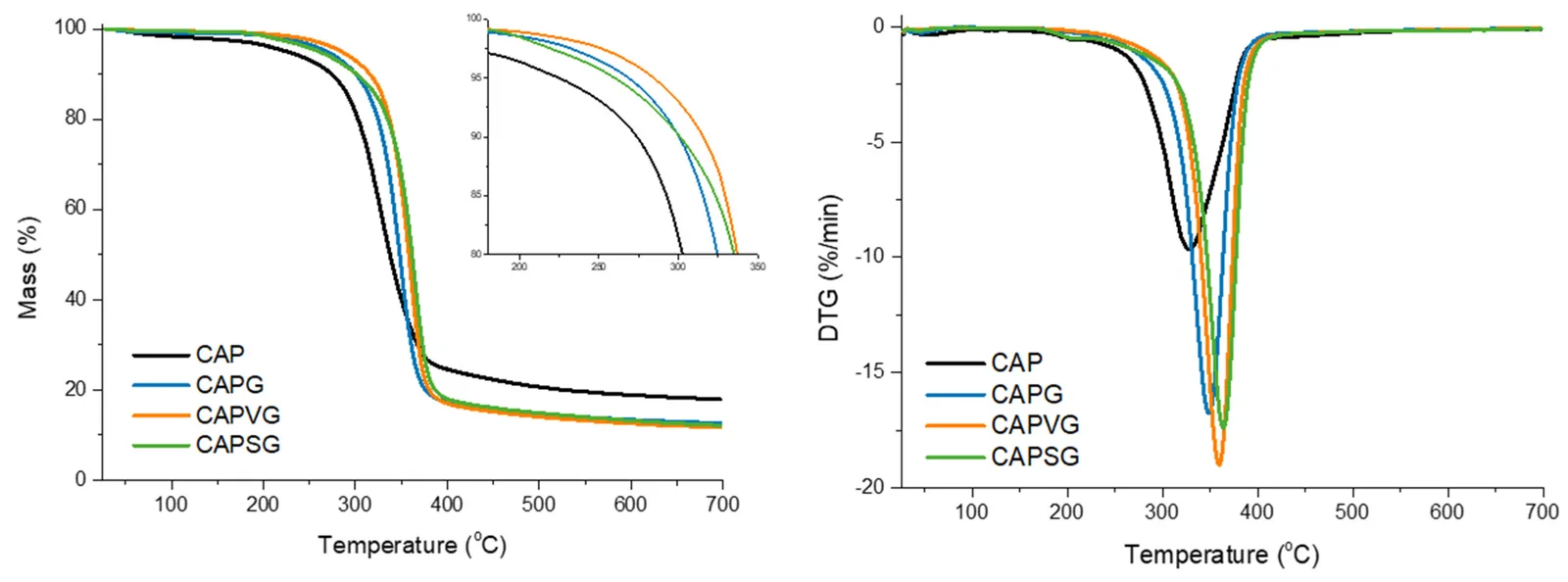
TGA curves of the synthesized membranes.

The thermal properties of the synthesized membranes were evaluated by means of DSC and TGA, and the corresponding results are presented in Figure 5 and Figure S2. The extracted thermal parameters are presented in Table 3.

The DSC data (Table 3) display a significant decrease in the enthalpy values of the low-temperature thermal event associated with the removal of absorbed water. The highest enthalpy (213 J/g) value recorded for this sample also indicates that a large amount of energy is associated with the removal of physically absorbed water [17,43]. This behavior suggests a high moisture retention in the neat CAP sample, most likely due to the abundance of polar functionalities (amino and hydroxyl) from both PEI and CA that tend to form hydrogen bonds with water. After GA crosslinking, the decrease in ΔH suggests reduced moisture retention due to the partial consumption of amino functionalities and the formation of a more compact network. Later in the process, the phenolic aldehyde-modified samples influence this behavior, with the vanillin-based membrane showing a markedly lower value for ΔH of 22 J/g while the salicylaldehyde shows an intermediate value of 88 J/g. The existence of two maximum temperatures associated with this process in the case of the CAPSG sample indicates the existence of different moisture domains within the material or possible overlap with a relaxation phenomenon.

Recent studies on the thermal properties of CA demonstrated that above 220 °C the acetyl bond starts to break and that the processing technique for the polymeric material may also strongly influence the thermal behavior [44]. The incorporation of PEI into the cellulose acetate matrix is expected to promote hydrogen-bonding interactions, which may contribute to improved structural stability of the membrane [45]. This may be the main reason for the relatively good thermal stability in the temperature domain between 50 and 200 °C. Thus, the recorded T_d3%_ temperature corresponds to this domain (50–200 °C) and can be associated with the removal of the physically absorbed water [46]. This reveals that the initial CAP sample tends to release absorbed water easily (~180 °C) compared with the aldehyde-modified samples. All samples exhibited a major degradation stage between 327 and 365 °C, corresponding predominantly to the decomposition of the cellulose acetate matrix, while the degradation of the PEI-rich phase is expected to partially overlap within this temperature range. However, it can be observed that, along with the inclusion of the aldehydes, the Tmax gradually increases, suggesting enhanced thermal resistance [37,47].

**Figure 6:**
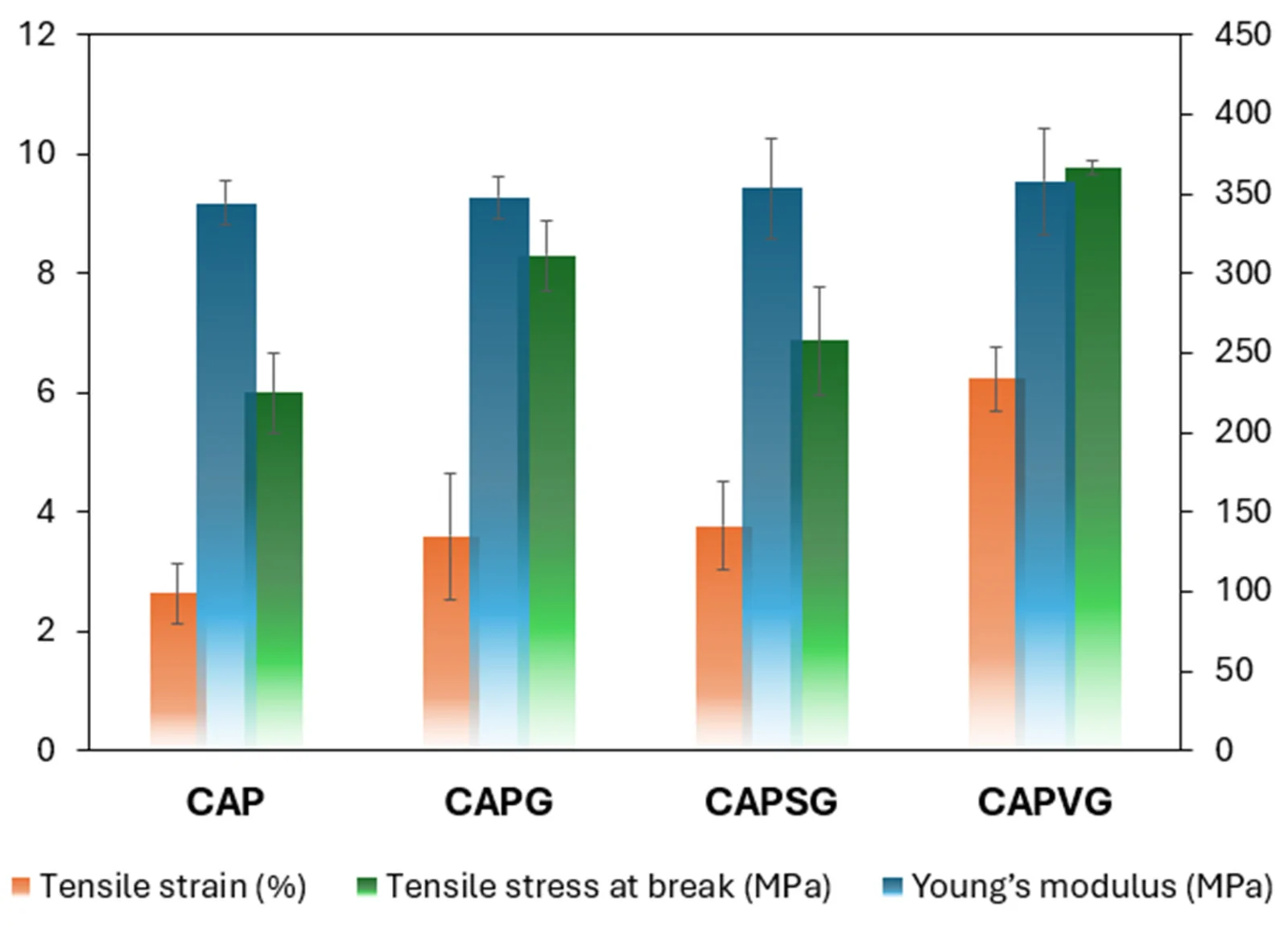
Mechanical parameters determined for the synthesized membranes.

The results of the mechanical property tests (Figure 6) show that the aldehyde modification influences the tensile response of the CA/PEI based membranes. The tensile strain and stress at break for the CAPG, CAPSG, AND CAPVG samples are generally higher compared with the CAP sample, suggesting improved ductility and mechanical resistance. The Young’s modulus shows only a slight increase, which demonstrates that the stiffness of the membranes is preserved. The best overall mechanical response was observed in the case of the CAPVG sample. In this case, one can conclude that these enhanced properties can arise from the combined contribution of imine bond formation, hydrogen bonding, and aromatic reinforcement effects. Although SAL and VAN have similar chemical structures containing aromatic aldehyde and phenolic functionalities, the latter compound includes an additional methoxy group, which may provide additional interactions [31,48]. This functional group can also contribute to an enhanced compatibility with the CAP matrix, leading to superior mechanical response [49].

### 3.3. Morphological Characterization

**Figure 7:**
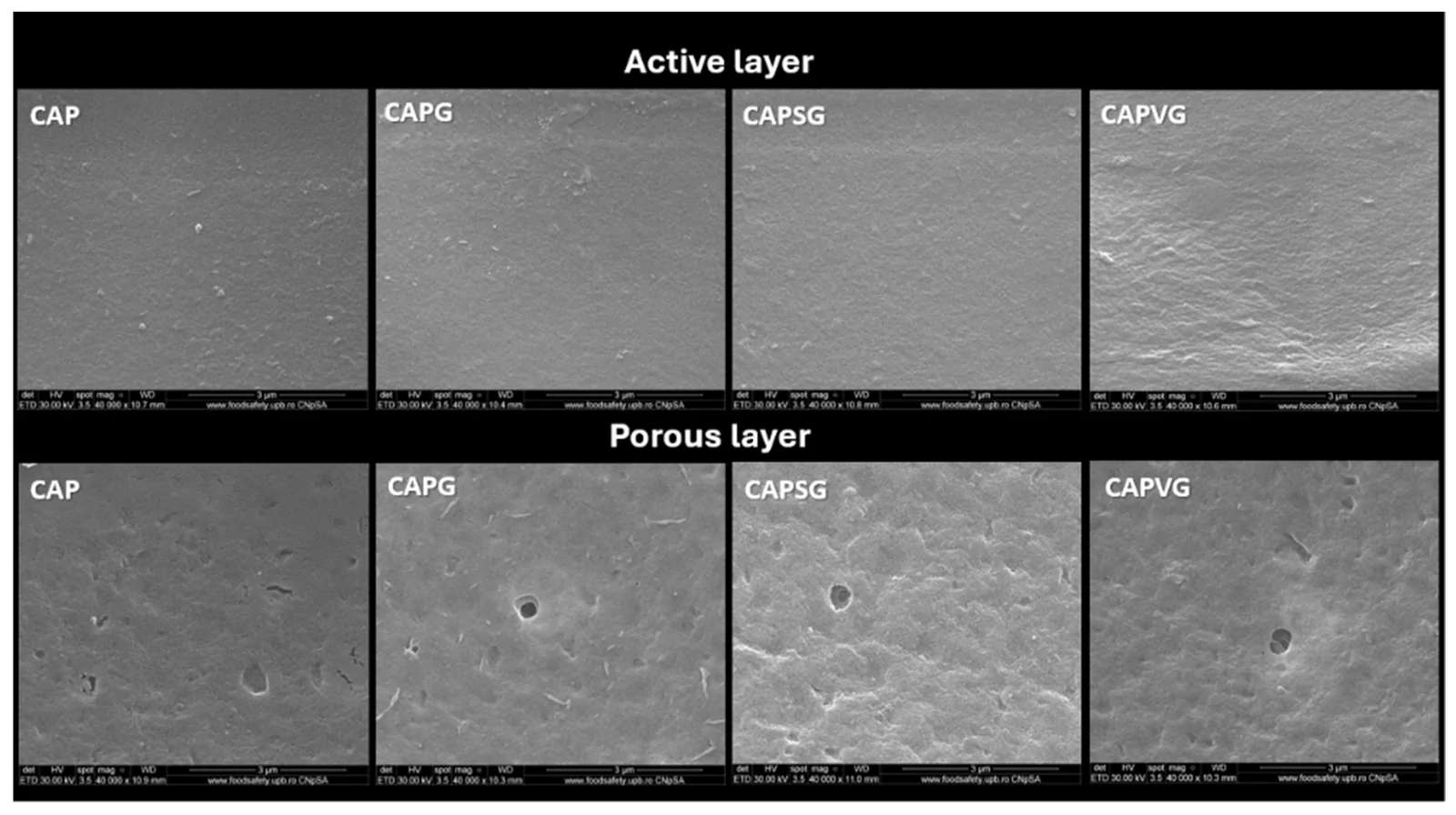
SEM micrographs of the active and porous layers of the synthesized membranes.

SEM analysis was used to evaluate the morphology of both active and porous layers of the CAP-based membrane and its aldehyde derivatives. Micrographs of the synthesized membranes are presented in Figure 7. The active layer of all samples displays a compact and relatively continuous appearance [50], while the porous side shows a heterogeneous and rough morphology [51]. After the inclusion of the aldehyde moiety, slight changes in surface texture can be observed, suggesting that the chemical crosslinking and modification influence the organization of the polymeric matrix without causing the formation of cracks or surface collapse.

The porous side of the membranes is also influenced by the aldehyde crosslinking and functionalization, showing visible changes in pore appearance [24]. This may be attributed to the additional interactions that may take place between the aldehyde, phenolic aldehyde, CA, and PEI that may influence the membranes’ microstructure.

**Figure 8:**
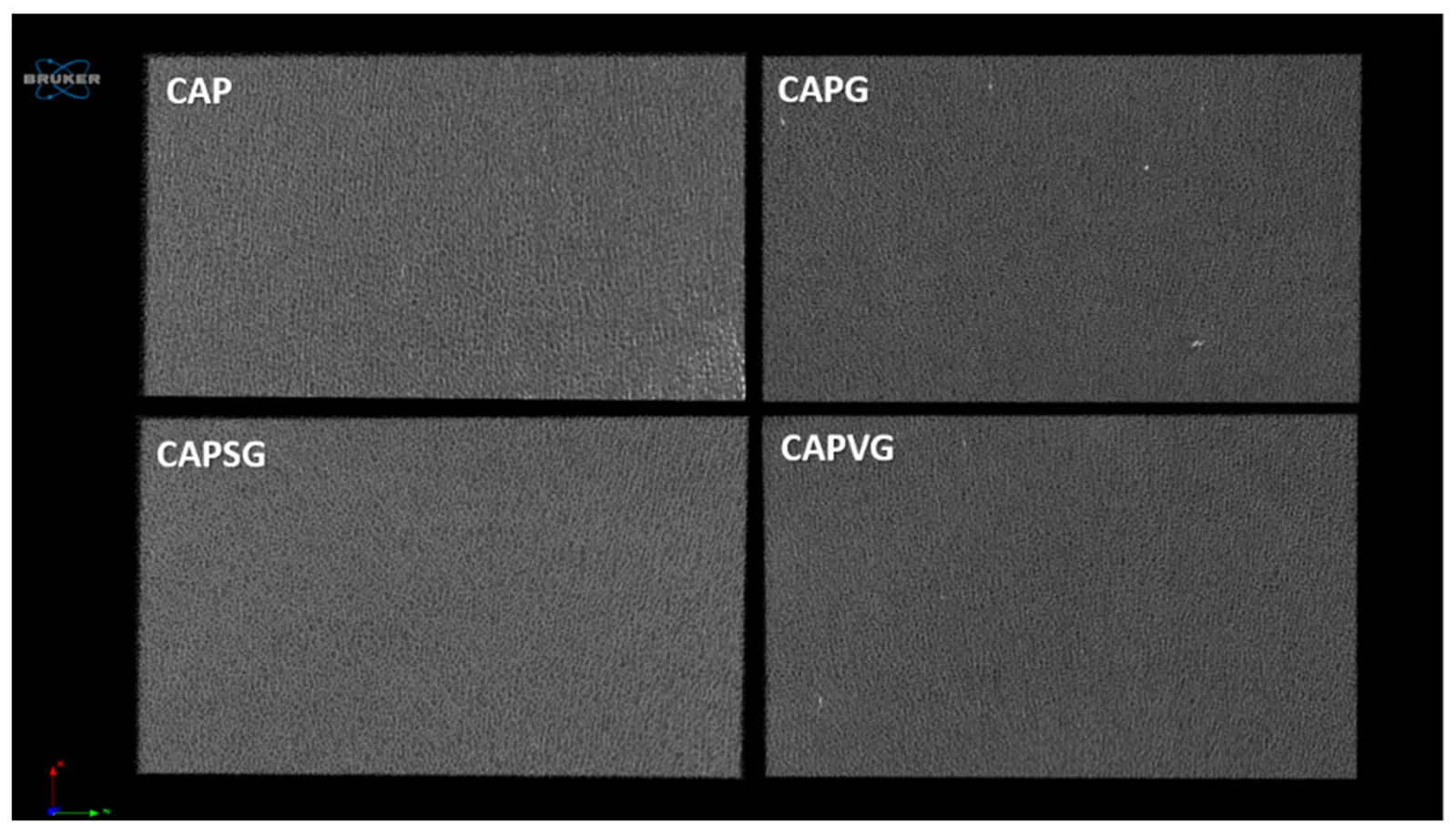
Micro-CT scans of the synthesized membranes.

Micro-CT analysis was employed to assess the 3D porous architecture of the synthesized membranes, and the corresponding results are presented in Figure 8 and Table 4.

It can be observed that the porosity of the membranes gradually increases along with the aldehyde inclusion, indicating the formation of a more open porous architecture. A similar trend can also be observed for the wall thickness, which slightly increased from 9.9 to 11.1 µm. This is a strong indicator of the preservation of the porous walls and may indicate a reinforcing effect induced by the aldehyde-induced interactions or partial crosslinked network [52,53]. Among the aldehyde-modified samples, CAPVG showed the highest porosity while maintaining a relatively consistent pore-size distribution. Along with the increased pore wall thickness, this may indicate a more balanced porous architecture. The improved mechanical and thermal properties of CAPVG may be associated with stronger interactions within the polymeric matrix forming the pore walls, leading to enhanced structural integrity.

The thermo-mechanical behavior of the modified membranes can be directly correlated with their microstructural characteristics. Although CAPVG exhibited the highest porosity, micro-CT analysis revealed a homogeneous pore distribution and a well-preserved interconnected structure, indicating that the increase in porosity did not compromise the mechanical integrity of the membrane. This observation is consistent with the improved mechanical performance of CAPVG, suggesting that the enhanced intermolecular interactions introduced by vanillin compensated for the higher void fraction. In contrast, the slightly denser structure of CAPSG may have contributed to its superior metal-ion retention by providing a higher density of accessible adsorption sites, despite showing less pronounced mechanical improvement. The comparable thermal stability of the modified membranes further indicates that the observed differences in mechanical behavior are primarily related to changes in the organization of the polymer network rather than significant variations in their thermal degradation mechanisms. Overall, these results demonstrate that membrane performance is governed by the combined effects of chemical modification and porous architecture rather than by porosity alone.

These findings highlight that the chemical structure of the phenolic modifier not only determines the availability of functional groups for metal binding but also influences the organization of the porous network, thus governing the balance between mechanical stability and separation performance.

### 3.4. Membrane Performance

Evaluation of membrane performance was mainly focused on comparing the CAPVG and CAPSG samples to assess the influence of the phenolic aldehyde structures vanillin and salicylaldehyde on metal-ion retention. The CAP membrane was not included in the filtration tests due to the water solubility and potential leaching of PEI, which could compromise membrane stability and lead to unreliable retention results.

**Figure 9:**
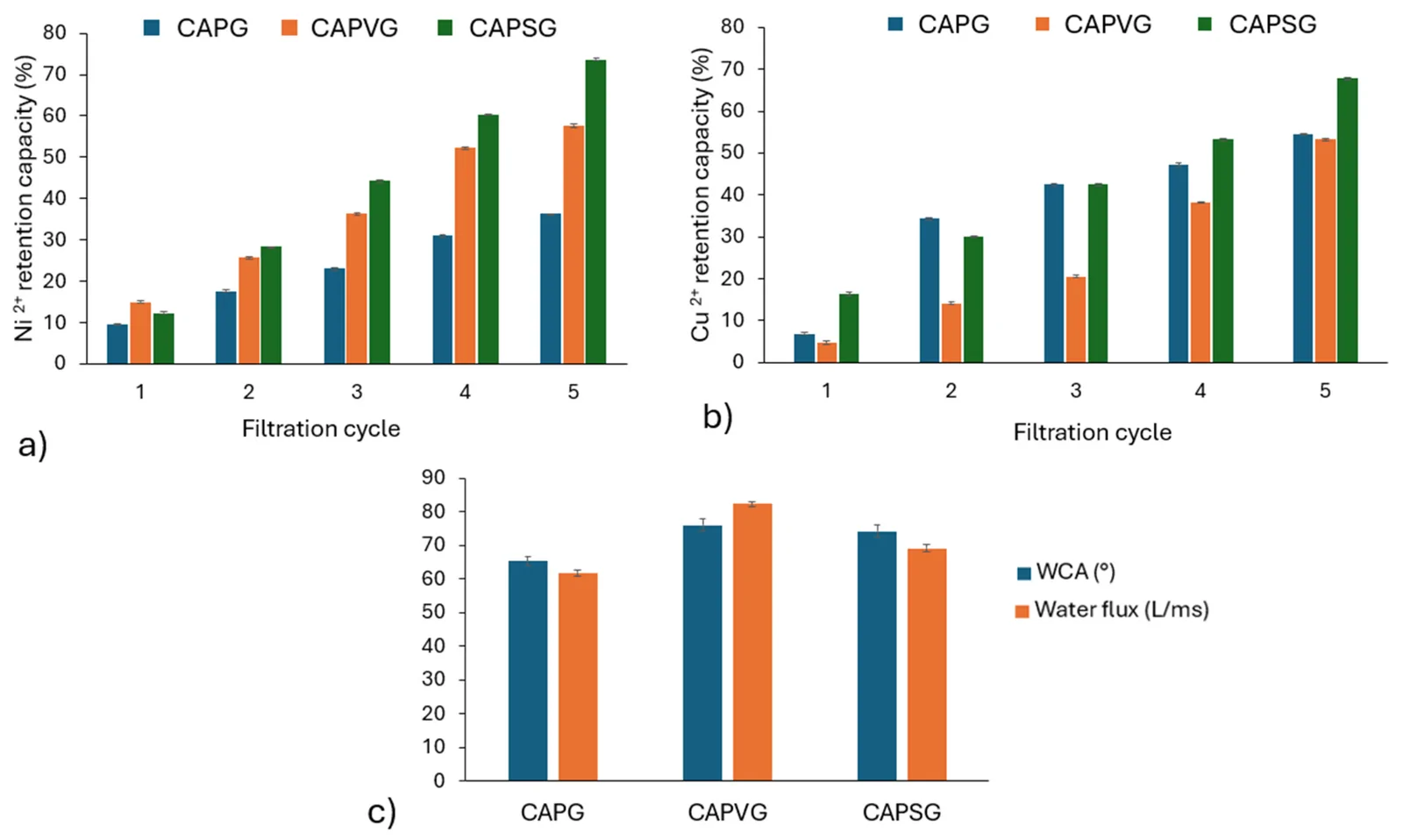
Ni^2+^ (**a**) and Cu^2+^ (**b**) retention efficiency of the aldehyde-modified CA/PEI membranes over five filtration cycles; water flux and water contact angles (WCA) for CA/PEI membranes (**c**).

Therefore, the ability of the phenolic aldehyde-modified membranes to retain metallic ions was evaluated over five consecutive filtration cycles against Cu^2+^ and Ni^2+^ ions; the corresponding results are presented in Figure 9.

The filtration test results revealed a gradual increase in metallic-ion retention over the five cycles. This may suggest that the active sites involved in ion binding become progressively accessible during repeated filtration, as well as progressive membrane conditioning during filtration. In the case of the evaluation of Ni^2+^ ion retention, the CAPSG membrane demonstrated the best performance, increasing from ~12% in the first cycle to more than 70% in the last cycle. The CAPVG membrane also showed an improvement in Ni^2+^ ion retention compared with CAPG, reaching around 58% in the fifth filtration cycle. A similar trend was also observed for the retention of Cu^2+^, wherein CAPSG again exhibited the highest final retention of up to ~70%.

Water contact angle measurements revealed that all samples display a hydrophilic character (WCA 65–76 °) even after crosslinking. This suggests a partial consumption of the amino groups from the PEI backbone during the crosslinking process as well as network densification. Nevertheless, water flux displays a conduct strongly related to the natural phenol used for the modification. Thus, the highest water flux was found with the CAPVG sample. This suggests that, in this case, the internal architecture of the membrane favors water transport.

The superior retention performance of the CAPSG sample may be associated with the chemical structure of salicylaldehyde. In this case, the adjacent position of the phenolic OH and aldehyde moieties can favor the formation of an N/O-type coordination environment for metallic-ion binding, although the exact coordination geometry requires further investigation. Overall, the metal-ion retention of the synthesized membranes may be governed by the combined contribution of the amine, imine, and oxygen-containing functionalities present in the system.

**Figure 10:**
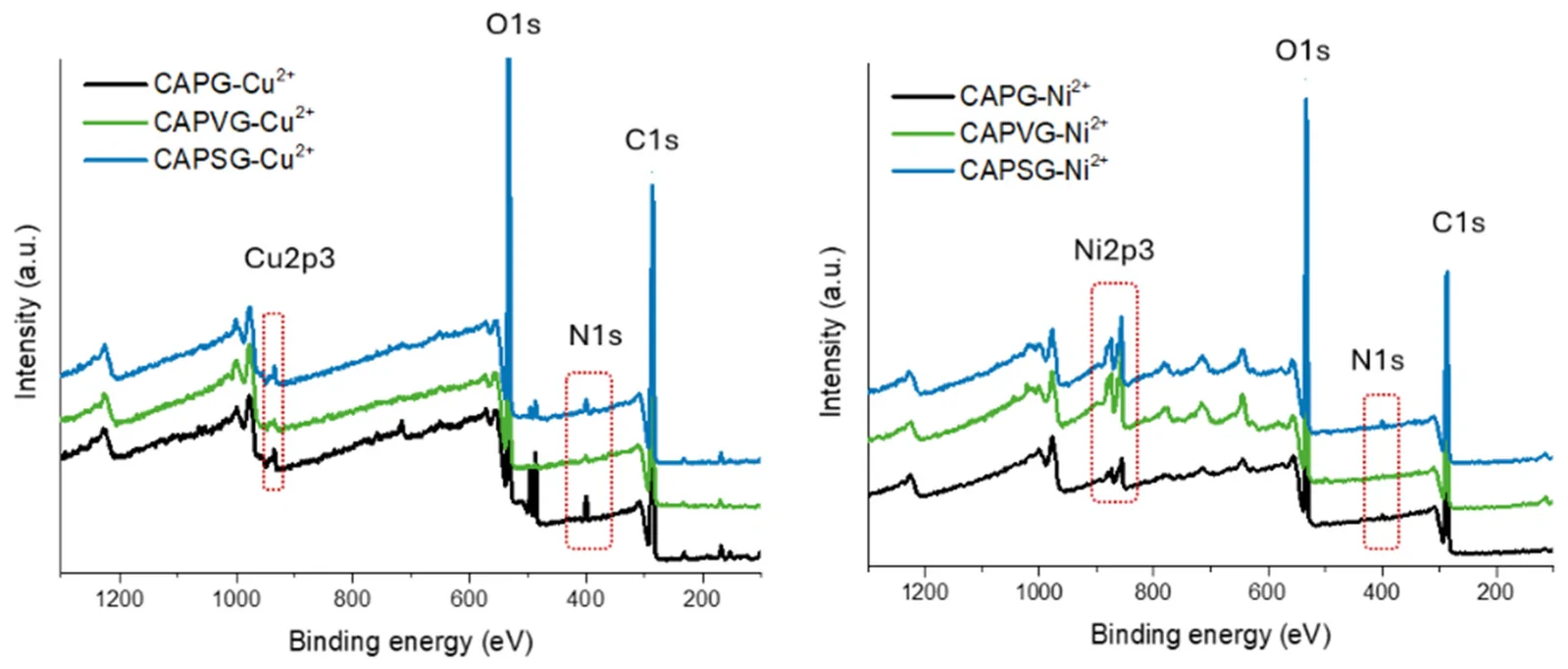
XPS Survey of the aldehyde-functional membranes after metal-ion retention.

XPS was further employed to validate the presence of the Ni^2+^ and Cu^2+^ ions on the surface of the aldehyde-modified membranes after filtration experiments. The survey spectra presented in Figure 10 confirm the presence of metallic ions on the surface of the membranes. The elemental composition data in Table 5 correlate with the filtration studies showing the highest amount of both Ni^2+^ and Cu^2+^ with the CAPSG membrane (2.76 and 0.98%).

**Figure 11:**
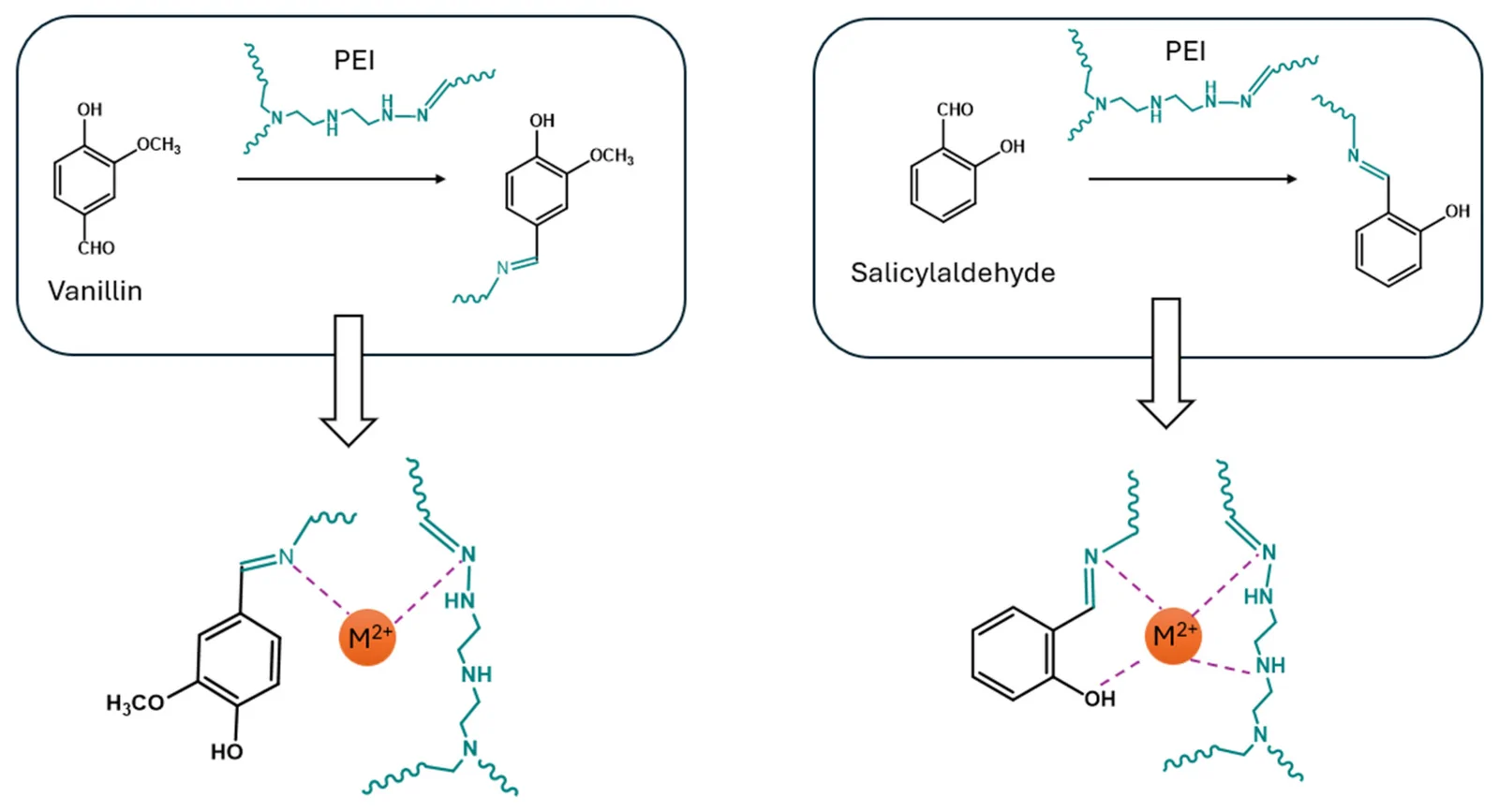
Schematic representation of the proposed metallic ions retention mechanism.

The superior adsorption performance of the salicylaldehyde-modified membrane can be explained by the different spatial arrangement of the functional groups introduced after Schiff base formation. In salicylaldehyde, the phenolic hydroxyl group is located in the ortho position relative to the imine linkage, allowing the formation of adjacent nitrogen- and oxygen-containing donor sites capable of acting cooperatively during metal coordination. Such an arrangement may facilitate the formation of stable N,O-chelate structures with transition metal ions, particularly Ni^2+^ and Cu^2+^ [54,55]. In contrast, the methoxy substituent of vanillin and the para-positioned hydroxyl group do not provide the same favorable geometric arrangement for bidentate coordination, although they may contribute to hydrogen bonding and increased matrix cohesion. Consequently, the higher retention observed for the salicylaldehyde-modified membrane is consistent with the more favorable coordination environment generated by its molecular structure. Schematic representation of the proposed metallic ions retention mechanism is presented in Figure 11.

## 4. Discussion

The present study highlights that the introduction of bio-based phenolic aldehydes provides an effective strategy for tailoring the properties of CA/PEI membranes. FTIR and XPS results indicate that membrane modification is mainly governed by the reaction between the aldehyde groups of vanillin and salicylaldehyde and the amino groups of PEI, while extensive hydrogen bonding contributes to stabilization. A similar strategy has been reported for amino-containing polymeric systems functionalized with aromatic aldehydes, wherein the simultaneous formation of imine bonds and secondary intermolecular interactions improves the stability of the final material [56,57]. The slight increase in water contact angles after crosslinking, together with the reduced moisture-related thermal events observed by DSC and TGA, indicates a moderate decrease in the availability of hydrophilic amino groups. Despite this, all membranes remained hydrophilic and exhibited increased water flux. This behavior suggests that permeation was governed predominantly by the internal porous architecture rather than by surface wettability alone. The micro-CT analysis supports this interpretation, revealing that the interconnected porous structure was preserved after modification, allowing efficient water transport despite the formation of a more compact polymer network.

The comparison between vanillin and salicylaldehyde further illustrates how subtle differences in molecular structure influence membrane performance. Although both aldehydes react with PEI through Schiff base formation, their substituent arrangement leads to distinct intermolecular interactions. The additional methoxy group of vanillin increases molecular polarity and may promote stronger hydrogen-bonding interactions within the polymer matrix, contributing to the improved mechanical behavior observed for the CAPVG membrane.

Overall, the present work extends previous studies on PEI-functionalized cellulose-based membranes, where heavy metal adsorption has been primarily attributed to the abundance of amino groups introduced by PEI. Here, the results demonstrate that the chemical structure of the phenolic modifier represents an additional design parameter capable of simultaneously influencing the organization of the polymer network, thermo-mechanical behavior, surface wettability, and heavy-metal adsorption. Consequently, the rational selection of bio-based aldehydes provides a simple yet effective strategy for optimizing cellulose-based adsorptive membranes for water purification applications.

## 5. Conclusions

In the present study, novel functional membranes were developed based on cellulose acetate, branched polyethyleneimine, and bio-sourced phenolic aldehydes. The influence of the chemical structure of the phenols was evaluated in terms of chemical structure and interactions, porous architecture, thermal stability, mechanical response, and metal-ion retention performance.

The aldehyde-mediated modification was investigated through FTIR and XPS analyses, which indicated changes in the chemical environment of the CA/PEI matrix consistent with Schiff base formation, PEI–aldehyde interactions, and the presence of oxygen- and nitrogen-containing functional groups.

Thermal analysis showed a reduced low-temperature DSC enthalpy and lower initial TGA mass loss after aldehyde treatment, suggesting decreased moisture retention due to partial amino group consumption and network densification. Mechanical testing revealed improved tensile behavior for the modified membranes, with the vanillin-containing sample showing the best balance between tensile strain, stress at break, and Young’s modulus. In contrast, the salicylaldehyde-modified membrane exhibited the highest metal-ion retention, reaching approximately 73% for Ni^2+^ and 67% for Cu^2+^ after five filtration cycles. This behavior was attributed to the formation of favorable N/O-type coordination environments associated with the ortho-phenolic structure of salicylaldehyde. SEM and micro-CT analyses confirmed the preservation of an asymmetric porous membrane architecture, with a compact active layer and a rougher, porous support side, while the modified membranes maintained high porosity values of approximately 82–83%.

Overall, this study demonstrates that structurally related phenolic aldehydes can be used to tune the chemical, morphological, thermal, mechanical, and metal-binding properties of CA/PEI membranes, offering a promising strategy for designing functional adsorptive membranes for water purification.

## Figures and Tables

**Table 1:** Sample abbreviation and composition description in DMF solution (10 g).

Sample Name	Sample Composition				
	CA (g) *	PEI (g) *	GA (mL) **	VAN (g) **	SAL (g) **
CAP	1.20	0.24	-	-	-
CAPG	1.20	0.24	0.94	-	-
CAPVG	1.20	0.24	0.94	0.76	-
CAPSG	1.20	0.24	0.94	-	0.61

*—in DMF solution with a total mass of 10 g; **—in 250 mL water–ethanol (70:30) solution.

**Table 2:** Elemental composition and quantification of surface analysis from XPS data.

Sample	C at.%	O at.%	N at.%	C–C/C–O
CAP	64.47	35.53	1.01	0.91
CAPG	65.11	33.56	1.33	0.67
CAPVG	63.25	36.11	0.64	0.98
CAPSG	65.43	34.16	0.41	0.54

**Table 3:** DSC and TGA thermal parameters extracted for the synthesized membranes.

Sample	ΔH (J/g)	Tmax (°C) *		Td3% (°C)	Td5% (°C)	Tmax (°C) **	Residual Mass (%)
CAP	213.60	52.7		182.4	224.7	327.2	17.87
CAPG	66.66	41.3		241.8	268.6	348.3	12.64
CAPVG	22.12	46.0		260.8	284.6	358.9	12.12
CAPSG	84.49	29.0	51.0	229.5	259.9	363.5	11.71

* data from DSC; ** data from TGA.

**Table 4:** Micro-CT structural parameters for the synthesized membranes.

Sample	Porosity (%)	St Th (µm)	St Sp (µm)	Obj/s (mm^−1^)
CAP	79.54	9.90	27.00	357
CAPG	82.49	11.00	31.00	323
CAPSG	82.86	11.00	31.00	321
CAPVG	83.08	11.00	35.00	354

St Th—pore wall thickness; St Sp—pore diameter; Obj/s—surface/volume ratio.

**Table 5:** Surface elemental composition (at.%) of CAPG, CAPSG, and CAPVG membranes after Cu^2+^ and Ni^2+^ retention determined by XPS.

Sample	O 1s (%)	C 1s (%)	N 1s (%)	Cu 2p3 (%)	Ni 2p (%)
CAPG-Cu^2+^	31.78	60.24	3.15	0.81	-
CAPSG-Cu^2+^	31.09	64.30	2.39	0.98	-
CAPVG-Cu^2+^	35.44	62.14	1.32	0.32	-
CAPG-Ni^2+^	34.40	63.07	0.63	-	1.26
CAPSG-Ni^2+^	33.68	62.35	1.20	-	2.76
CAPVG-Ni^2+^	35.82	60.20	0.93	-	2.73

## Data Availability

The original contributions presented in this study are included in the article. Further inquiries can be directed to the corresponding author.

## Supplementary Materials

The following supporting information can be downloaded at https://www.mdpi.com/article/10.3390/polym18182193/s1, Figure S1: XPS Survey and N1s deconvoluted peak of the synthesized membranes; Figure S2: DSC thermograms of synthesized membranes.
