# Supplementary material for: Bio-Based Phenolic Aldehyde Functionalization of Cellulose Acetate–Polyethyleneimine Membranes for Enhanced Ni2+ and Cu2+ Retention
Source: Polymers (Basel). 2026 Sep 8;18(18):2193. doi: 10.3390/polym18182193 (PMC13610607; doi:10.3390/polym18182193)
Supplement: Supplementary file 1 [file polymers-18-02193-s001.zip › polymers-4542222-supplementary.pdf]

Bio-Based Phenolic Aldehyde Functionalization of CA/PEI Membranes for Enhanced Ni^{2+} and Cu^{2+} Retention

Eduard Ionut Piscanu [1] ,Celina Maria Damian [2], Andreea Madalina Pandele [1,2] , Madalina Oprea [1,2], Adrian Ionut Nicoara [3] and Stefan Ioan Voicu [1,2,4,*]

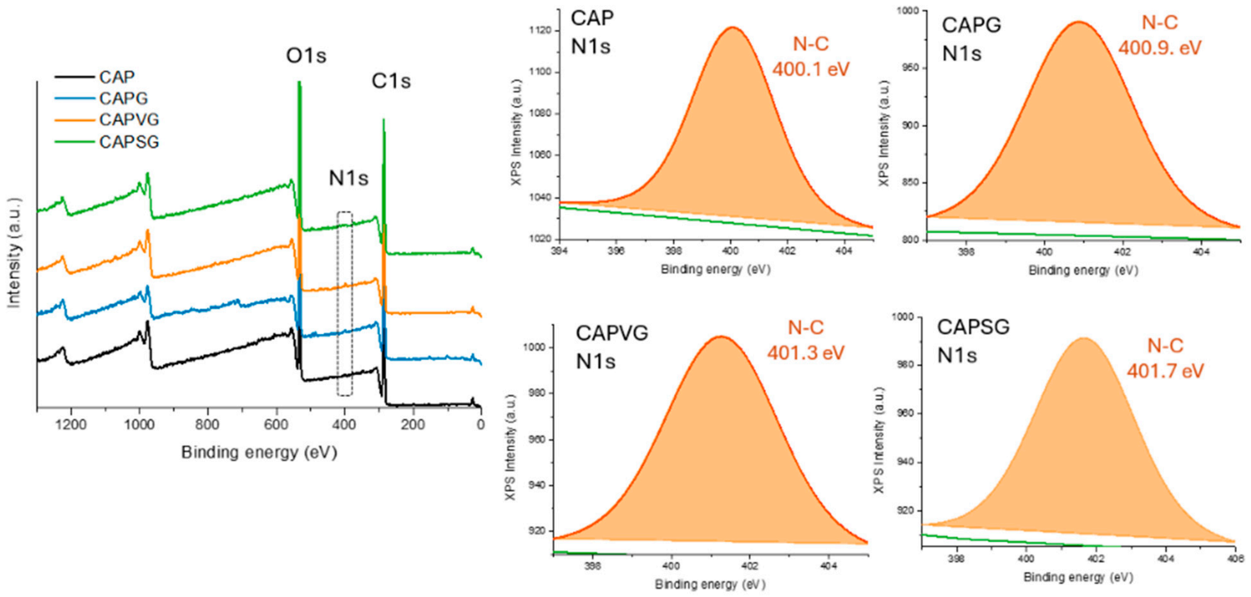

Figure S1. XPS Survey and N1s deconvoluted peak of the synthesized membranes.

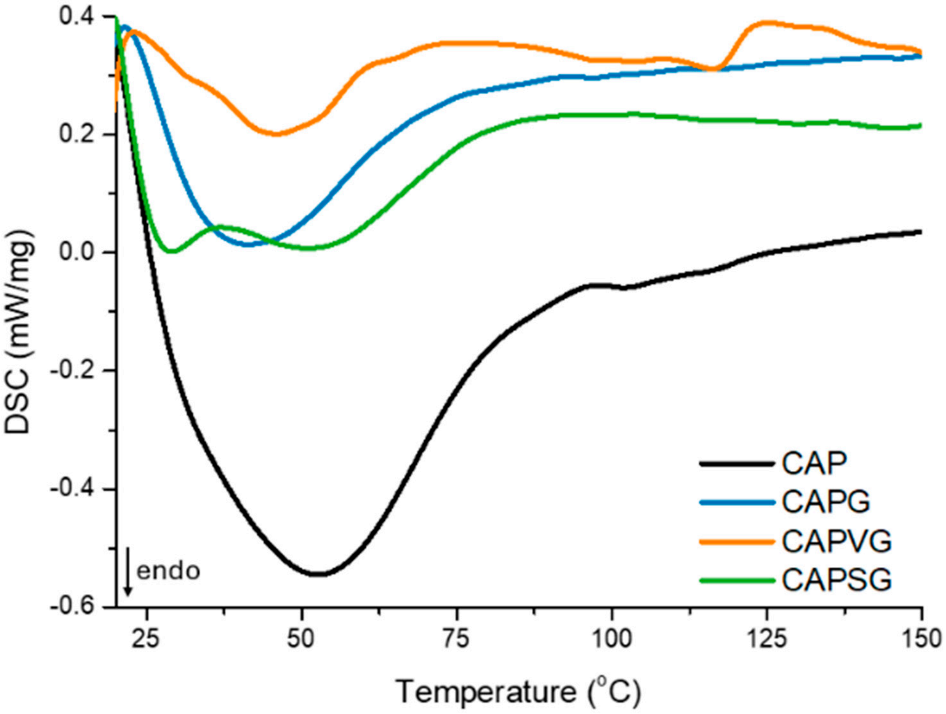

Figure S2. DSC thermograms of synthesized membranes.
